# Optimization of the marinating conditions of cassava fish (*Pseudotolithus* sp.) fillet for Lanhouin production through application of Doehlert experimental design

**DOI:** 10.1002/fsn3.285

**Published:** 2015-09-18

**Authors:** Janvier Mêlégnonfan Kindossi, Victor Bienvenu Anihouvi, Générose Vieira‐Dalodé, Noël Houédougbé Akissoé, Djidjoho Joseph Hounhouigan

**Affiliations:** ^1^ Department of Nutrition and Food ScienceFaculty of Agronomic SciencesUniversity of Abomey‐Calavi01 BP 526CotonouBenin

**Keywords:** Citric acid, fish fillet, Lanhouin, marination, optimization, salt

## Abstract

Lanhouin is a traditional fermented salted fish made from the spontaneous and uncontrolled fermentation of whole salted cassava fish (*Pseudotolithus senegalensis*) mainly produced in the coastal regions of West Africa. The combined effects of NaCl, citric acid concentration, and marination time on the physicochemical and microbiological characteristics of the fish fillet used for Lanhouin production were studied using a Doehlert experimental design with the objective of preserving its quality and safety. The marination time has significant effects on total viable and lactic acid bacteria counts, and NaCl content of the marinated fish fillet while the pH was significantly affected by citric acid concentration and marination duration with high regression coefficient *R* ^2^ of 0.83. The experiment showed that the best conditions for marination process of fish fillet were salt ratio 10 g/100 g, acid citric concentration 2.5 g/100 g, and marination time 6 h. These optimum marinating conditions obtained present the best quality of marinated flesh fish leading to the safety of the final fermented product. This pretreatment is necessary in Lanhouin production processes to ensure its safety quality.

## Introduction

Lanhouin is described as traditional fermented salted fish obtained from the spontaneous and uncontrolled fermentation of whole salted fish (Anihouvi et al. 2007, 2012; Kindossi et al. 2012). The ripening is one of the steps of Lanhouin process which has a significant effect on flesh quality, and consequently on consumer acceptance. Ripening increases the aroma and the softness of the flesh as a result of enzymatic hydrolysis and breakdown of proteins into peptides and amino acids, due to microbial and chemical activities (Anihouvi et al. 2007, 2009). It was found that the level of histamine in the final product was higher than the level accepted under European legislation (Anihouvi et al. 2012, 2006; Commission Regulation (EU), 2013). Moreover, the marination process is proposed as a step which combined the ripening and salting steps in Lanhouin production process with the aim of reducing the pH and pathogen bacteria growth and enhancing the sanitary quality of the final product.

The marination process is a commonly used method for preservation of fish flesh, meat, and vegetable through the simultaneous action of salt and organic acids. It involves an increase in ionic strength and decrease in pH (Poligne and Collignan 2000; Goli et al. 2011; Komoltri and Pakdeechanuan 2012). It was observed that the process allowed to increase the product yield, the reduction in pH and water loss, and the improvement of tenderness of meat (Komoltri and Pakdeechanuan 2012). Marination preserves fish by using sodium chloride and organic acid solutions (Sallam 2008). The basic function of marinating preservation is associated with the synergic activity of organic acids and salt from marinade to the changes in fish fillets. Organic acids and salt are added to the fish not only to retard the microbiological and enzymatic activity, but also to change the taste and textural properties of the fish, resulting with a semipreserved product with limited shelf life (Poligne and Collignan 2000; Goli et al. 2011). Fish fillets soaking in acidic marinades causes on one hand a transfer of salt ions, acid to the fish, with a reduction in pH, and on the other hand leakage of the fish components (Adams and Nout 2001; Baygar et al. 2010; Theron and Rykers Lues 2011).

The objective of this study was to investigate the best marinating variables (acid and salt concentrations, marinating time) and to determine their effects on the physicochemical and microbiological properties of fish fillet. In order to reduce the number of experiments, a Doehlert design was used. Doehlert matrices (Doehlert 1970) present many advantages such as its spherical experimental domain with uniformity in space filling, its ability to explore the whole domain, and its potential to allow for sequential study where the experiments can be reused when the boundaries have not been well chosen at first (Bensalah et al. 2010). The methodologies of the experimental design and response surface analysis were used to check the effect of the three variables.

## Materials and Methods

### Fish and marinade

Cassava fish (*Pseudotolithus* sp.) was purchased from Cotonou seaport and transported in an icebox with dry ices to the laboratory. The fish was washed, scaled, gutted, headed, and again washed twice before filleting. The weight of each fish was approximately 40 g. The marinade solutions were prepared from extrapure citric acid (Scharlau Chemie S.A., EC Label, Spain European Union), sodium chloride (NaCl) (GPR rectapur 11G130020 EC Label, European Union), and distilled water.

### Experimental setup and methods

Batches of three fish fillets (120 g) were immersed in 300 mL plastic bowls containing 250 mL of marinade. Each duplicate batch corresponding to one combination of sodium chloride × citric acid × marination time was shaked manually, each for 30 min at an ambient temperature of 30 ± 2°C during 4, 6, and 8 h of marination. After treatment, the fish fillets were removed from the marinade slowly to surface, dried, and then used for physicochemical and microbiological analysis. Experiments were carried out according to the Doehlert experimental design for modeling and graphic representation of various responses studied and statistical analysis of the effect of the factors (sodium chloride, citric acid, marination time). The Doehlert (uniform network of three factors) experimental design was carried out in marinade containing water, NaCl (0–20 g/100 g of water), and citric acid (0–5 g/100 g of water). The marination durations were from 4 to 8 h. Kinetics were carried out in marinades comprising water, NaCl (0, 5, 10, 15, and 20 g/100 g of water), and citric acid (0.33, 1.06, 1.78, 2.50, 3.22, 3.94, and 4.67 g/100 g of water) with analyses carried out after 4, 6, and 8 h of marination. These immersions were done in the same experimental setup as for the Doehlert plan. The responses measured were pH, chloride, TVC (total viable count), and LAB (lactic acid bacteria).

### Experimental design

A Doehlert uniform shell design (Doehlert 1970) was applied to find the best conditions of the marinating process for Lanhouin processing. Correlations with the response variables were established with a quadratic model (Equation):

$$Y=a_{o}+\sum_{i}a_{i}X_{i}+\sum_{i}a_{ii}X_{i}^{2}+\sum_{ij}a_{ij}X_{i}X_{j}.$$

where *Y* is a response (pH, chloride, TVC, LAB), *X*_*i*_ is a variable (NaCl, citric acid, marination time), *a*_*o*_ is the constant of the model, *a*_*i*_ is the linear regression coefficient, *a*_*ii*_ is the quadratic regression coefficient of *X*_*i*_, and *a*_*ij*_ is the regression coefficient of the interaction between *X*_*i*_ and *X*_*j*_.

Marination was carried out in ternary (acid + salt + water) and binary (acid + water or salt + water) solutions (Table 1).

**Table 1:** Experimental range and levels of the variables (Doehlert matrix)

Independent variables	Codes	Variables levels						
NaCl (g/100 g)		−1	−0.5	0	0.5	1		
	X1	0	5	10	15	20		
Citric acid (g/100 g)		−0.866	−0.577	−0.288	0	0.288	0.577	0.866
	X2	0.33	1.06	1.78	2.5	3.22	3.94	4.67
Marination time (h)		−0.816	0	0.816				
	X3	4	6	8				

### Physicochemical analysis

pH of the samples was measured with a pH meter (Hanna Instrument HI 9318) according to the NF V 04‐108 (AFNOR, 1974) method. Sodium chloride content (NaCl) was determined by measuring the chloride ion concentration with a chloride analyzer (Corning MKII model 926; Sherwood Scientific Ltd., Cambridge, U.K.) after extraction in 0.3 N nitric acid.

### Microbiological analysis

Ten (Sallam 2008) grams of each Lanhouin sample were introduced aseptically in a sterile Stomacher bag and 90 mL of sterile diluent containing 0.1% peptone (Oxoid L37, Basingstoke, Hampshire, England), 0.8% sodium chloride (NaCl) (Merck KGaA, Darmstadat, Germany) with pH adjusted to 7.2 was added. The mixture was then homogenized for 2 min using a Stomacher bag (Lab‐Blender, Model 80, Seward Medical, London, U.K.) (ISO 6887‐1, 1999). One milliliter of the suspension was serially used for microbial counts according to the ISO norms.

Total viable counts were enumerated using PCA (Plate Count Agar, Oxoid CM0325) and PCA plates were incubated at 30°C for 72 h (ISO 4833, 2003). LAB were enumerated using de Man, Rogosa, Sharpe agar (MRS, Oxoid CM0361) and the MRS plates were incubated at 30°C for 72 h (ISO 15214, 1998). *Enterobacteriaceae* were enumerated using Violet Red Bile Glucose Agar (VRBG, Oxoid, CM0485) and the plates were incubated at 37°C for 24 h (ISO 21528‐2, 2004). *Staphylococcus aureus* and coagulase‐positive *Staphylococcus* were enumerated using Baird Parker agar base (Oxoid CM0275) supplemented with egg yolk tellurite emulsion (SR54, Basingstoke, Hampshire, England). The inoculated plates were incubated at 37°C for 24 h (ISO 6888, 1999; ISO 7937, 2004).

### Statistical analysis

STATISTICA (version 7.1, Stat Soft France, 2006) was employed for regression analysis of the data and for estimation of the coefficients of the regression equation. The statistical significance of the model was determined by the Fisher's test through ANOVA (analysis of variance). The canonical analysis was also carried out to predict the shape of the curve generated by the model.

## Results and Discussion

Responses of the dependent variables (pH, NaCl content, TVC, and LAB) obtained from the model for marinating conditions for the improvement of Lanhouin quality are presented in Table 2. *Enterobacteriaceae* and *Staphylococcus aureus* detected were lower than 1 log cfu/g in all samples. The results of the multiples regression analysis which provided the estimates of the model coefficients are listed in Table 3.

**Table 2:** Responses (pH, NaCl, TVC, and LAB) obtained from the experimental matrix through application of Doehlert design methodology

Experiment	Coded values			Experimental values			Responses			
	*X*_1_	*X*_2_	*X*_3_	NaCl (g/100 g of water)	Citric acid (g/100 g of water)	Marination time (h)	pH	NaCl (g/100 g)	TVC (log cfu/g)	LAB (log cfu/g)
1	0.5	0.288	0.816	15	3.22	8	4.28	8.8	5.4	3.2
2	−0.5	−0.288	−0.816	5	1.77	4	4.00	5.6	6.8	2.4
3	−0.5	−0.866	0	5	0.33	6	5.27	5.9	7.0	3.9
4	0	−0.577	0.816	10	1.05	8	4.91	8.9	6.5	3.0
5	0.5	−0.866	0	15	0.33	6	5.16	10.5	7.1	3.8
6	−0.5	0.577	−0.816	10	3.94	4	3.39	6.3	6.0	2.7
7	0	0	0	10	2.5	6	4.13	7.8	6.3	4.3
8	1	0	0	20	2.5	6	4.09	10.8	5.4	3.0
9	−1	0	0	0	2.5	6	3.91	0.7	6.1	3.1
10	0.5	0.866	0	15	4.66	6	3.50	8.9	4.9	1.8
11	−0.5	0.866	0	15	4.66	6	3.43	7.6	4.9	2.8
12	0.5	−0.288	−0.816	15	1.77	4	3.83	8.5	6.7	2.9
13	−0.5	0.288	0.816	15	3.22	8	3.64	6.8	5.2	3.2

The experimental values (*U*_*i*_) were calculated from the coded values (*X*_*i*_) using the formula: *U*_*i*_ = *U*_0*i*_ + *X*_*i*_Δ*U*_*i*_, where *U*_0*i*_ is the centered value and Δ*U*_*i*_ the range. For NaCl, $U_{01}=\frac{10g}{100g}$ and $\Delta U_{1}=\frac{10g}{100g}$; for citric acid, $U_{02}=\frac{2.50g}{100g}$ and $\Delta U_{2}=\frac{2.49g}{100g}$; for marination time, *U*_03_ = 6 h and Δ*U*_2_ = 2 h.

**Table 3:** Regression coefficients of the variable in the model and their corresponding *R* ^2^

Coefficient^1^	pH	NaCl (g/100 g)	TVC (log cfu/g)	LAB (log cfu/g)
*a*_o_	4.132	7.771	6.261	4.333
Linear				
*a*_1_	0.099	3.894**	−0.127	−0.111
*a*_2_	−0.971**	−0.244	−1.212**	−0.624**
*a*_3_	0.329*	0.811	−0.504*	0.274
Quadratic				
*a*_11_	−0.131	−2.005	−0.517	−1.279*
*a*_22_	0.317	1.219	−0.214	−1.234*
*a*_33_	−0.235	−0.247	−0.066	−1.531**
Interaction				
*a*_12_	0.106	−1.920	−0.063	−0.552
*a*_13_	0.461	0.090	0.201	−0.054
*a*_23_	−0.150	0.877	−0.218	0.093
*R*²	0.83	0.75	0.75	0.57

^1^Indices 1, 2, and 3 refer, respectively, to the variables of salt ratio, citric acid concentration, and marination time. $Y=a_{0}+a_{1}X_{1}+a_{2}X_{2}+a_{3}X_{3}+$ où *X*_1_ = salt ratio (g/100 g), *X*_2_ = citric acid concentration (g/100 g), *X*_3_ = marination time (h), **coefficients significant at *P* ≤ 0.01, *coefficients significant at *P* < 0.05.

### Effect of salt rate, citric acid concentration, and marination duration on the pH

**Figure 1:**
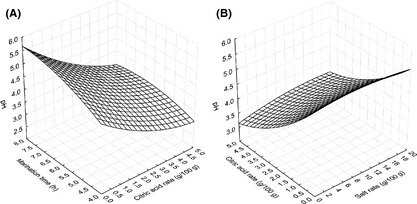
pH values of marinated fish fillet affected by the process variables. (A) Citric acid concentration and marination time (salt rate = 10 g/100 g of water). (B) Citric acid concentration and salt rate (marination time = 6 h).

pH of marinated fish fillet (between 3.39 and 5.16) (Table 2) were significantly lower than the pH of raw fish fillet (6.94). The table of regression coefficient (Table 3) revealed that the pH of fish fillet was negatively and significantly (*P* ≤ 0.01) affected by the linear effect of citric acid (*X*_2_) concentration and positively affected (*P* < 0.05) by the linear effect of duration of marination (*X*_3_). The model explained 80% of the variations in pH (Table 3). Figure 1 shows the evolution of the pH of the marinating fish fillets affected by the process variables. This pH value of the fish fillet marinated is one of the criteria for determining not only the acceptability of marinated products, but also their stability and preservation (Kopermsub and Yunchalard 2008). The study showed that the optimal conditions required, with the decrease in pH of the fish fillet during marination, were marination time of 4–8 h and citric acid concentration of 0–5 g/100 g with 10 g/100 g salt ratio (Fig. 1A).

Also, when the marination time was held constant, the pH value decreased linearly with citric acid concentration and salt rate (Fig. 1B). This observation could be due to the fact that at 6 h and considering the citric acid concentration, the pH value decreased as the acid level of the marinade increased. At these conditions, pH values of the marinated fish fillet are low and desirable for the final product. These acid conditions were unbearable for the activity of the microorganisms.

### Effect of salt rate, citric acid concentration, and marination duration on NaCl

**Figure 2:**
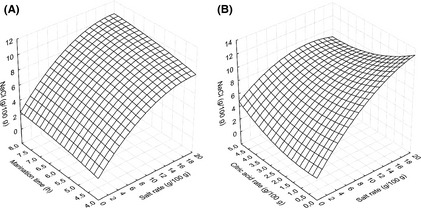
NaCl content of marinated fish fillet affected by the process variables. (A) Salt rate and marination time (citric acid = 2.5 g/100 g of water). (B) Citric acid concentration and salt rate (marination time = 6 h).

Response surface analysis and the regression coefficient for NaCl content are presented in Figure 2 and Table 3, respectively. NaCl content in marinated fish fillet was significantly and negatively (*P* ≤ 0.01) affected by linear effect of salt rate (*X*_1_). The model explained 75% of the variations in NaCl (Table 3). From Figure 2A it was observed that when the citric acid concentration was kept constant at the central point, the NaCl content increased slowly with marination time and salt rate. But with marination time at constant, the increase in NaCl content in fish fillet was affected by the immigration of aqueous NaCl contained in the brine with a low concentration of citric acid (Fig. 2B). From this study, the optimal conditions required to have an optimal NaCl concentration of the fish fillet were the duration of marination (6 h), the concentration of citric acid (0–2.5 g/100 g), and the salt rate (0–20 g/100 g).

### Effect of salt rate, citric acid concentration, and marination duration on total viable count and lactic acid bacteria

**Figure 3:**
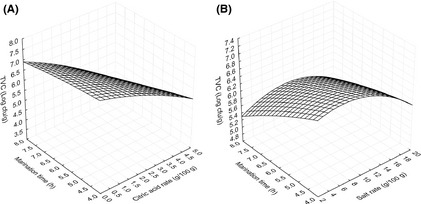
Total viable count of marinated fish fillet affected by the process variables. (A) Citric acid concentration and marination time (salt rate = 10 g/100 g of water). (B) Salt rate and marination time (citric acid = 2.5 g/100 g of water).

Total viable count changes in fish fillet during marinating were negatively and significantly affected by linear effects of citric acid (*X*_2_) (*P* ≤ 0.01) and duration of marination (*X*_3_) (*P* < 0.05). The model explained 75% of the variation in TVC (Table 3). Figure 3 demonstrated TVC changes in fish fillet during marination. TVC decreased progressively with citric acid concentration and marination time when the salt rate was remained constant (Fig. 3A). Also when the citric acid concentration was held constant at the central point, TVC were relatively stable with salt rate and marination time (Fig. 3B).

**Figure 4:**
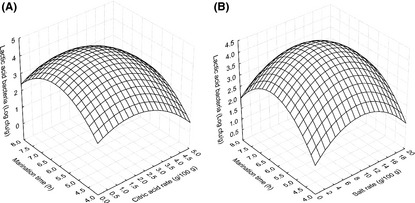
Lactic acid bacteria of marinated fish fillet affected by the process variables. (A) Citric acid concentration and marination time (salt rate = 10 g/100 g of water). (B) Salt rate and marination time (citric acid = 2.5 g/100 g of water).

Lactic acid bacteria changes in fish fillet during marinating were negatively and significantly (*P* ≤ 0.01) affected by linear effects of citric acid (*X*_2_). The negative quadratic effects of salt ratio, citric acid concentration, and duration of marination were highly significant (*P* < 0.05) on LAB. The model explained 57% of the variation in LAB (Table 3). Figure 4 demonstrated LAB changes in fish fillet during marination. LAB increased with citric acid concentration and duration of marination when salt rate was held at 10 g/100 g of salt ratio (Fig. 4A). It could be explained that, as the marination time was prolonged, LAB had more time to grow with the increase in acid content in the fish flesh. However, when the citric acid concentration was held constant, the LAB decreased with salt rate and marination time (Fig. 4B). This observation could be due to the fact that at 2.5 g/100 g of citric acid, the increase in NaCl content in the fish flesh slowly decreased the water availability and consequently reduced the growth of LAB.

Other microorganisms such as *Enterobacteriaceae* and *Staphylococcus aureus* were determined, but the load was lower than 1 log cfu/g.

In marinating process, the raw fish fillets were preserved in citric acid and salt to inhibit the proliferation of bacteria causing the putrefaction and those which are sensitive to salt and acid. Spoilage bacteria may still occur due to the growth of nonputrefactive organisms which can survive in acidic and salt environments. Therefore, LAB may grow in this condition.

### Checking of the model

**Figure 5:**
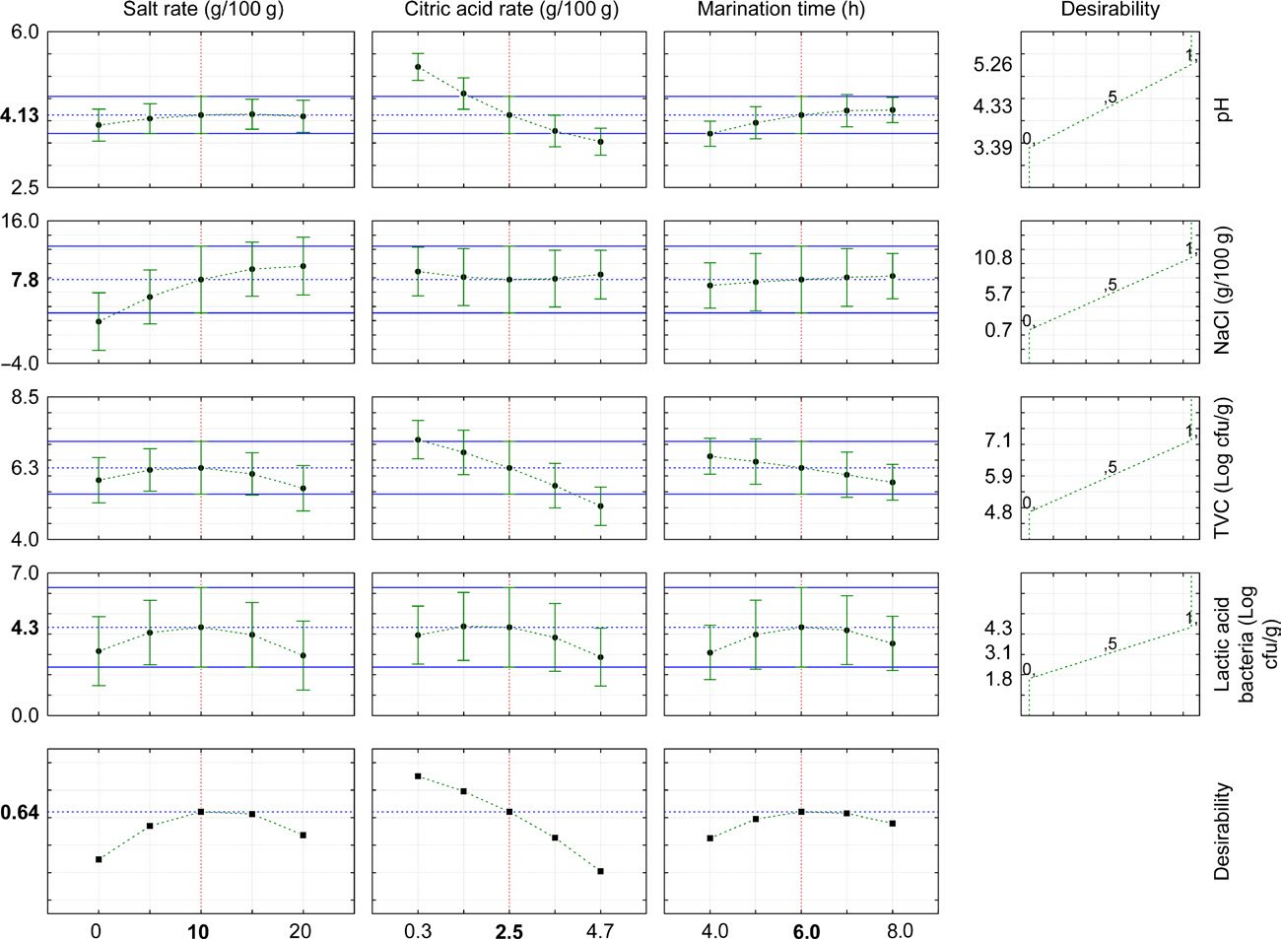
Predicted values and marinating condition desirability.

In order to check the model carried out, the desirability function was used to optimize the marination time, salt rate, and citric acid concentration. As indicated by the desirability function, the optimum conditions for marination are salt rate of 10 g/100 g, citric acid concentration of 2.5 g/100 g, and 6 h of marination time, resulting in pH value of 4.13, NaCl of 7.6 g/100 g, TVC of 6.2 log cfu/g, and LAB of 4.3 log cfu/g (Fig. 5). The predicted and experimental values for the pH, NaCl, TVC, and LAB are summarized in Table 4. The experimental and the predicted values of the marinated fish fillet are similar. The pH value of marinated fish fillet obtained is low under this condition and such a low pH value is desirable to create an unfavorable environment for the proliferation of the pathogenic microorganisms and consequently guarantee safety of the product. The final product from the application of the optimum condition of marination processing contained pH value of 4.43, NaCl of 9.8 g/100 g, TVCs of 4.2 log cfu/g, and LAB of 2.8 log cfu/g (Table 4). The pH values <4.5 and NaCl content >6.0 g/100 g might inhibit the growth of spoilage bacteria (Poligne and Collignan 2000; Sallam 2008). This pretreatment is necessary in Lanhouin production processes for European market to ensure safety and quality of the final product. Compared to raw fish fillet, this pretreatment resulted in lower pH of the muscle and significantly different NaCl content in the fillets.

**Table 4:** Predicted values and experimental values for the water content of the pH, NaCl, TVC, and LAB

Parameters	Limits	Desirability	Predicted values	Experimental values	
				Marinated fish fillet	Dried fermented salted product
pH	3.39–5.26	0.39	4.13	3.71 ± 0.1	4.43 ± 0.12
NaCl (g/100 g)	0.7–10.8	0.70	7.8	3.8 ± 0.9	9.8 ± 0.3
TVC (log cfu/g)	4.8–7.1	0.62	6.2	5.4 ± 0.5	4.2 ± 0.3
LAB (log cfu/g)	1.8–4.3	1.0	4.3	4.6 ± 0.1	2.8 ± 0.8

TVC, total viable counts; LAB, lactic acid bacteria.

## Conclusion

The use of response surface methodology allowed studying the simultaneous effect of salt rate, citric acid concentration, and marination duration and their interactions on product quality during marination of fish. The results showed that the salt ratio, citric acid concentration, and marination time affect the microbiological and chemical quality of marinated fish flesh significantly (*P* ≤ 0.05). The optimum marinating conditions based on desirable pH values in sample were established as salt ratio of 10 g/100 g, citric acid concentration of 2.5 g/100 g, and marination time of 6 h. Experimental values obtained from the optimum conditions were conformed to those predicted from the model. These conditions present the best quality of marinated fish flesh leading to the safety of the final fermented product.

## Conflict of Interest

None declared.
